# Rapid Versus Gradual Weaning of External Ventricular Drain: A Systematic Literature Review and Meta-analysis

**DOI:** 10.1007/s12028-023-01766-6

**Published:** 2023-06-12

**Authors:** Florian Ebel, Eric Lichter, Luigi Mariani, Raphael Guzman, Jehuda Soleman

**Affiliations:** 1 Department of Neurosurgery, University Hospital of Basel, Spitalstrasse 21, 4031 Basel, Switzerland; 2 Faculty of Medicine, University of Basel, Basel, Switzerland; 3 Department of Pediatric Neurosurgery, University Children’s Hospital of Basel, Basel, Switzerland

**Keywords:** External ventricular drain, Weaning, Ventriculoperitoneal shunt, Hydrocephalus

## Abstract

The insertion of an external ventricular drain (EVD) is one of the most common neurosurgical procedures. Whether the weaning method (gradual or rapid) influences the ventriculoperitoneal shunt (VPS) insertion rate has not been conclusively established. The aim of this study is to provide a systematic literature review and conduct a meta-analysis of studies comparing gradual with rapid EVD weaning regarding VPS insertion rate. Articles were identified by searching the Pubmed/Medline, Embase, and Web of Science databases throughout October 2022. Two independent researchers assessed the studies for inclusion and quality. We included randomized trials, prospective cohort studies, and retrospective cohort studies, which compared gradual and rapid EVD weaning. The primary outcome was VPS insertion rate, whereas secondary outcomes were EVD-associated infection (EVDAI) rate and length of stay in the hospital and intensive care unit (ICU). Four studies directly comparing rapid versus gradual EVD weaning, with 1337 patients suffering from subarachnoid hemorrhage, were identified and included in the meta-analysis. VPS insertion rate was 28.1% and 32.1% in patients with gradual and rapid EVD weaning, respectively (relative risk 0.85, 95% confidence interval 0.49–1.46, *p* = 0.56). Further, the EVDAI rate was comparable between the groups (gradual group 11.2%, rapid group 11.5%, relative risk 0.67, 95% confidence interval 0.24–1.89, *p* = 0.45), whereas length of stay in the ICU and hospital were significantly shorter in the rapid weaning group (2.7 and 3.6 days, respectively; *p* < 0.01). Rapid EVD weaning seems comparable to gradual EVD weaning concerning VPS insertion rates and EVDAI, whereas hospital and ICU length of stay is significantly reduced.

**Supplementary Information:**

The online version contains supplementary material available at 10.1007/s12028-023-01766-6.

## Introduction

External ventricular drain (EVD) insertion is one of the most common emergency procedures in neurosurgery. One of the leading indications for EVD is aneurysmal subarachnoid hemorrhage (aSAH), for which up to 87% of patients require an EVD to treat acute hydrocephalus [1–4]. In 17% of patients, a permanent cerebrospinal fluid (CSF) diversion in terms of a ventriculoperitoneal shunt (VPS) is required in the short or long term after EVD removal [5]. Because of high dysfunction rates of VPS, with a consecutive rate of revision surgery of 23.3% over 8 years, reducing the rate of VPS insertion is of high socioeconomic importance [6, 7]. Once the acute phase is overcome, the EVD should be removed to reduce the risk of infections, and it can be withdrawn in two different ways. Either the EVD is gradually weaned, thereby gently decreasing the amount of CSF delivered, or it is rapidly closed. Whether a rapid or gradual EVD weaning influences the need for VPS insertion has been the focus of research, although no clear consensus exists [8]. The aim of this systematic literature review and meta-analysis was to compare rapid and gradual EVD weaning protocols regarding the rate of VPS insertion, EVD-associated infections, and the length of stay (LOS) in the hospital and the intensive care unit (ICU).

## Methods

### Search Strategy and Inclusion Criteria

The systematic literature search was conducted following the updated PRISMA Guidelines 2020 [9]. The literature databases Pubmed/Medline, Embase, and Web of Science were searched, and corresponding studies were identified throughout 15/10/2022. We used a search string including the keywords “weaning” AND “EVD” (Supplementary Table 1). Two authors assessed all results independently (FE and EL) for eligibility using Rayyan [10]. When consensus opinion could not be reached, a third researcher was to be consulted (JS). The study was registered on PROSPERO (CRD42022367236). Randomized controlled trials (RCTs) and prospective and retrospective cohort studies comparing rapid and gradual EVD weaning were included. Studies reporting EVD weaning in children, without direct comparison between gradual weaning and direct clamping of EVD or published in a language other than English were excluded from this analysis. All included studies, except the study by Rao et al. [11], which included ten patients (6.6%) with angiogram-negative SAH, analyzed patients with aSAH. Because of the paucity of literature on this topic, an exception was made, and the study by Rao et al. [11] was nevertheless included in the analysis.

### Quality Assessment

The quality assessment for the included studies was performed independently by FE and EL. The risk of bias tool was used for RCTs, and the Newcastle–Ottawa Scale (NOS) and Robins-1 were used for retrospective and prospective cohort studies, respectively.

### Data Extraction

The primary outcome parameter was the need for permanent CSF diversion by a VPS insertion. Implantation of a VPS was necessary if the EVD could not be weaned due to clinical deterioration of the patient or if the EVD could be successfully removed initially but the patient developed hydrocephalus at a later stage. Secondary outcome parameters were the rate of EVD-associated infections (EVDAI), the total LOS in the hospital, and the LOS in the ICU.

Rapid weaning was defined as such if the EVD was closed immediately. On the other hand, gradual weaning was defined as slowly increasing the hydrostatic backpressure in steps of varying magnitude over a time period of a couple of days until the drain was finally closed.

### Statistical Analysis

A descriptive analysis of the baseline characteristics and primary and secondary outcomes for included studies was undertaken. The meta-analysis included only studies directly comparing rapid and gradual EVD weaning. The relative risk (RR) was used as the effect size to compare binary data, whereas the mean difference was used to compare continuous outcomes. Because of the heterogeneity between included studies, a random-effects model was applied. Forest plots were calculated and presented for all outcomes. All analyses were done using the SPSS Software (Version 28; IBM Corp., New York).

## Results

**Fig. 1:**
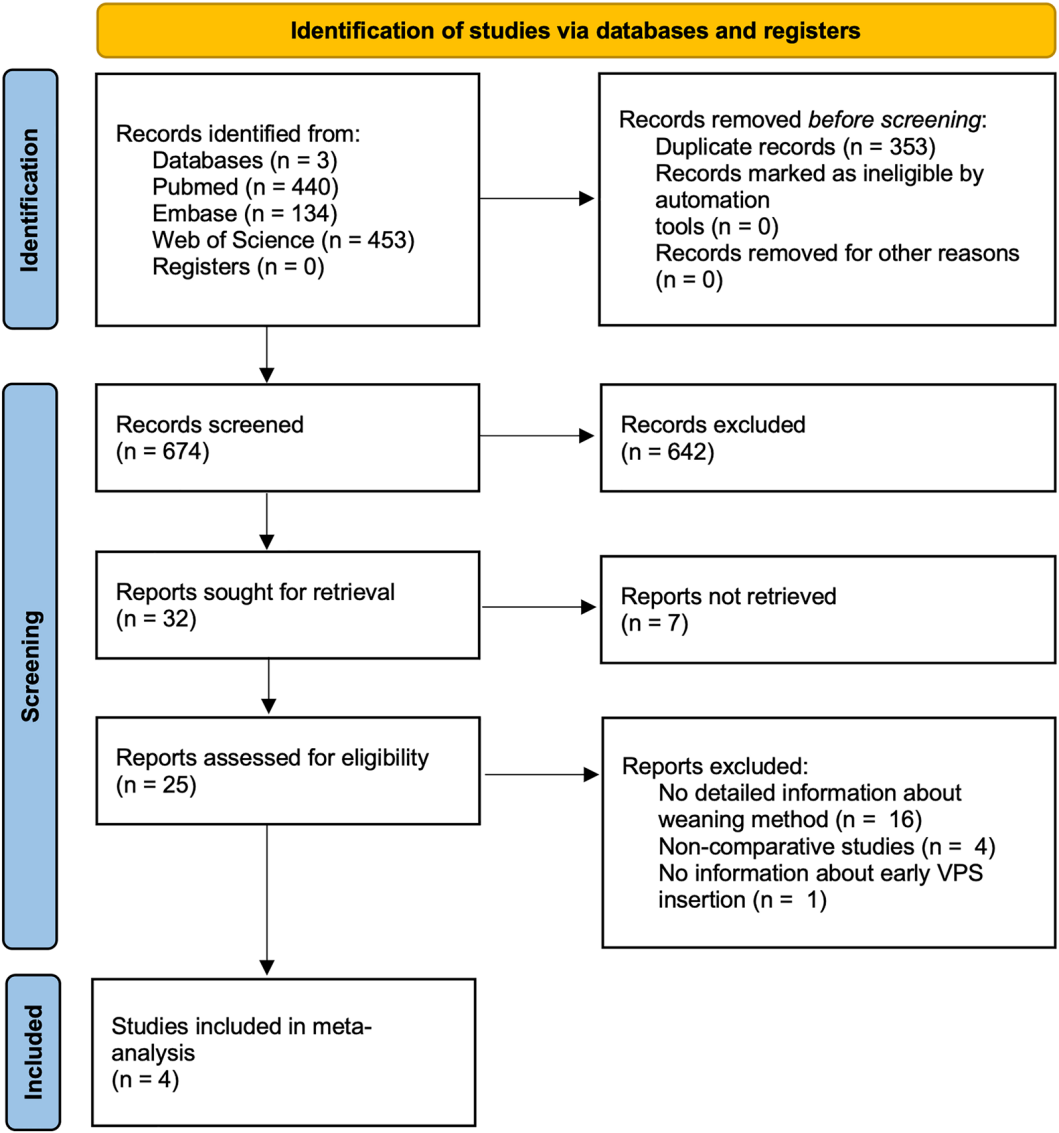
Flow chart of the number of studies identified in the systematic literature search and included in the analysis

After the initial search yielded 1027 articles, eight studies [11–18] analyzing EVD weaning were detected; however, four studies [15–18] were excluded from the analysis because they did not directly compare management protocols of EVD weaning. Finally, four studies were included in the meta-analysis (Fig. 1) [11–14]. One study (25%) was a prospective randomized trial [14], one study (25%) was a prospective cohort study [13], while two studies (25%) were retrospective cohort studies [11, 12] (Table 1). A total of 1,337 patients were included in the meta-analysis, of whom 695 (52%) and 642 (48%) received gradual and rapid EVD weaning, respectively [11–14]. A total of 35.4% and 32.4% of patients with gradual and rapid EVD weaning were men. The mean age was 56.2 and 55.9 years in the gradual and rapid weaning groups, respectively [11–14]. In 1327 (99.3%) patients, the etiology for CSF circulation disorder was aSAH, while in 10 (0.7%) patients, it was due to an angiogram-negative SAH (Table 1). Two (50%) studies reported the Glasgow Coma Scale at admission, with gradual and rapid weaning group reporting a mean Glasgow Coma Scale of 12.2 and 12.7, respectively [11, 13]. Time to EVD weaning was reported by only one study and was longer in patients with gradual EVD weaning, averaging 9.9 days compared with 8 days in patients with rapid EVD (Table 2) [13].

**Table 1:** Overview of included studies for meta-analysis

References	Type of study	Total	Indication for EVD		Weaning method		Definition of weaning method	
			aSAH	Angio-negative SAH	Gradual	Rapid	Gradual	Rapid
Rao et al. [11]	Retrospective CS	152	142 (93.4)	10 (6.6)	79 (52)	73 (48)	Gradual EVD rise by 5 cmH_2_O until 20 cmH_2_O, after which the EVD was closed	Immediate EVD closure
Jabbarli et al. [12]	Retrospective CS	965	965 (100)	–	510 (52.8)	455 (47.2)	Gradual EVD rise by 5 cmH_2_O every 24 h until 25 cmH_2_O, after which the EVD was closed	Immediate EVD closure
Chung et al. [13]	Prospective CS	139	139 (100)	–	66 (47.5)	73 (52.5)	Gradual EVD rise daily	Immediate EVD closure
Klopfenstein et al. [14]	Prospective RT	81	81 (100)	–	40 (49.4)	41 (50.6)	Gradual EVD rise by 5 cmH_2_O every 24 h until 25 cmH_2_O, after which the EVD was closed	Immediate EVD closure
Total		1337	1327	10	695	642		

All values presented as number (%) of patients related to the corresponding study, if not otherwise specified

*Angio-negative* angiogram-negative, *aSAH* aneurysmal subarachnoid hemorrhage, *CS* cohort study, *EVD* external ventricular drain, *RT* randomized trial

**Table 2:** Descriptive analysis of the four included studies regarding their baseline parameters and the primary and secondary end points

Parameter	No. of studies reported (%)	Total	Gradual weaning	Rapid weaning
No. of patients	4/4 (100) [11–14]	1,337	695 (52)	642 (48)
Baseline characteristics				
Men	4/4 (100) [11–14]	453 (33.9)	246 (35.4)	207 (32.4)
Age (years) (mean)	4/4 (100) [11–14]	56	56.2	55.9
GCS at admission (mean)	2/4 (50) [11, 14]	12.4	12.2	12.7
Time to weaning (days) (mean)	1/4 (25) [13]	9	9.9	8
Primary end point				
VPS insertion	4/4 (100) [11–14]	401 (30)	195 (28.1)	206 (32.1)
Secondary end points				
Vasospasms	2/4 (50) [11, 13]	106 (7.9)	55 (7.9)	51 (7.9)
EVD-associated infection	3/4 (75) [11–13]	152 (11.4)	78 (11.2)	74 (11.5)
LOS ICU (days) (mean)	3/4 (75) [11, 13, 14]	16.9	18.1	15.6
LOS total (days) (mean)	3/4 (75) [11, 13, 14]	22.7	24.1	21.2

All values presented as number (%) of patients if not otherwise specified

*EVD* external ventricular drain, *GCS* Glasgow Coma Scale, *ICU* intensive care unit, *LOS* length of stay, *No.* number, *VPS* ventriculoperitoneal shunt

### Ventriculoperitoneal Shunt Insertion

**Fig. 2:**
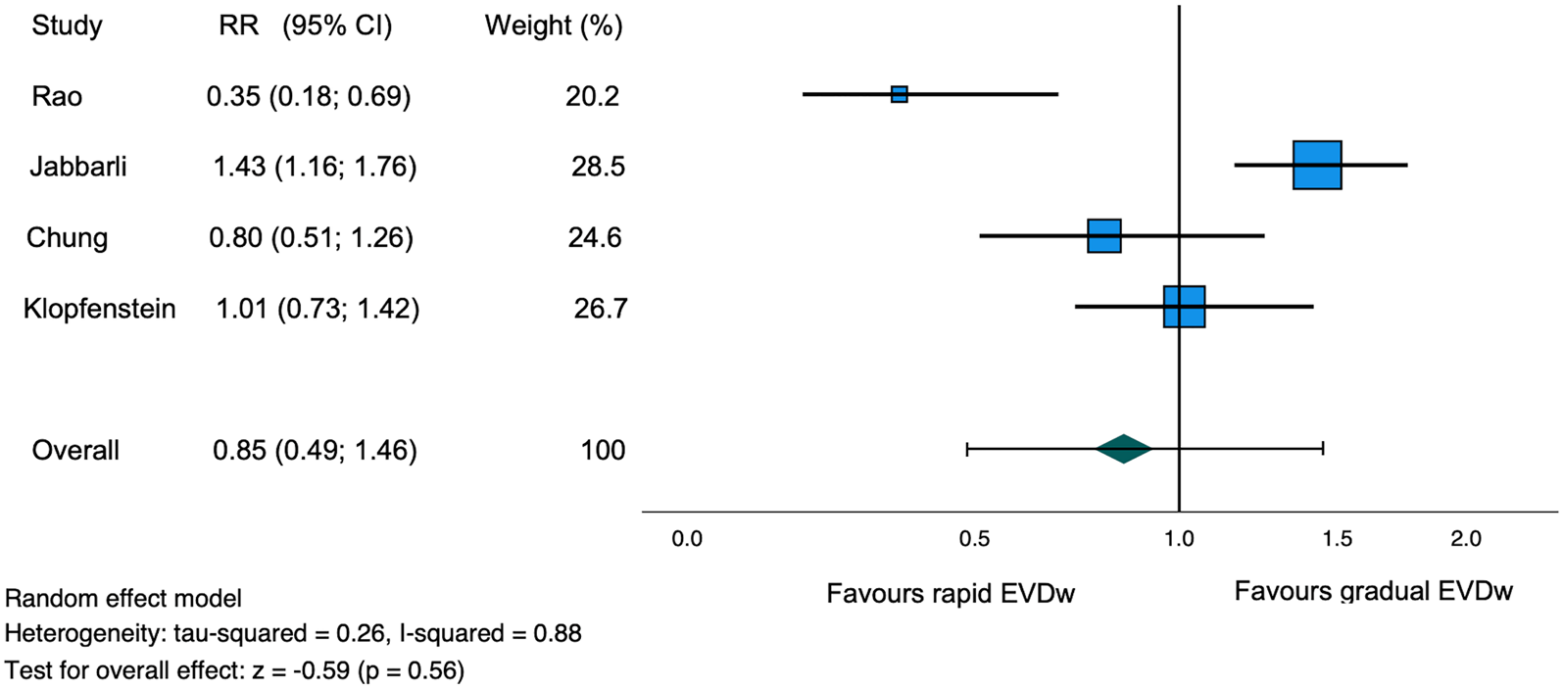
Forest plot of ventriculoperitoneal shunt insertion rate. *CI* confidence interval, *EVDw* external ventricular drain weaning, *RR* relative risk

The VPS insertion rate was reported in all studies [11–14]. Of the 1337 patients, 220 patients (16.5%) were derived from prospective studies and 1117 patients (83.5%) from retrospective studies [11–14]. A comparable VPS insertion rate was observed in the rapid and gradual weaning groups (32.1% and 28.1%, RR 0.85, 95% confidence interval [CI] 0.49–1.46, *I*^2^ = 88%, *z* =  − 0.59, *p* = 0.56, Table 2, Fig. 2).

### External Ventricular Drain-Associated Infection

**Fig. 3:**
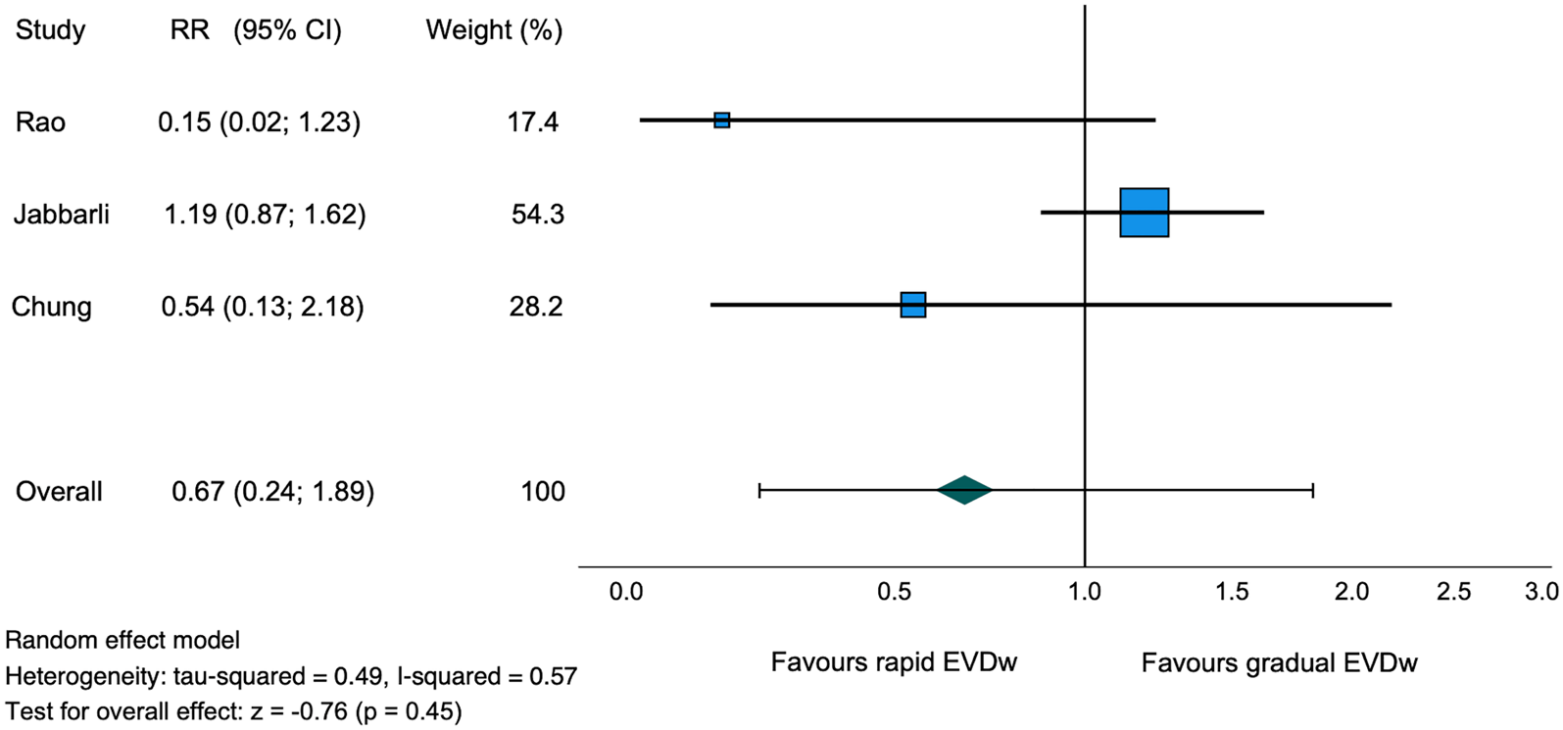
Forest plot of EVD-associated infection rate. *CI* confidence interval, *EVD* external ventricular drain, *EVDw* external ventricular drain weaning, *RR* relative risk

External ventricular drain–associated infections were reported in three studies (75%) with 1256 patients [11–13]. Of the 1256 patients, 1117 patients (88.9%) from the two retrospective studies and 139 patients (11.1%) from the prospective cohort study were included [11–13]. The EVDAI rate between the rapid and gradual weaning groups was comparable (11.5% and 11.2%, respectively, RR 0.67, 95% CI 0.24–1.89, *I*^2^ = 57%, *z* =  − 0.76, *p* = 0.45, Table 2, Fig. 3).

### Length of Stay

**Fig. 4:**
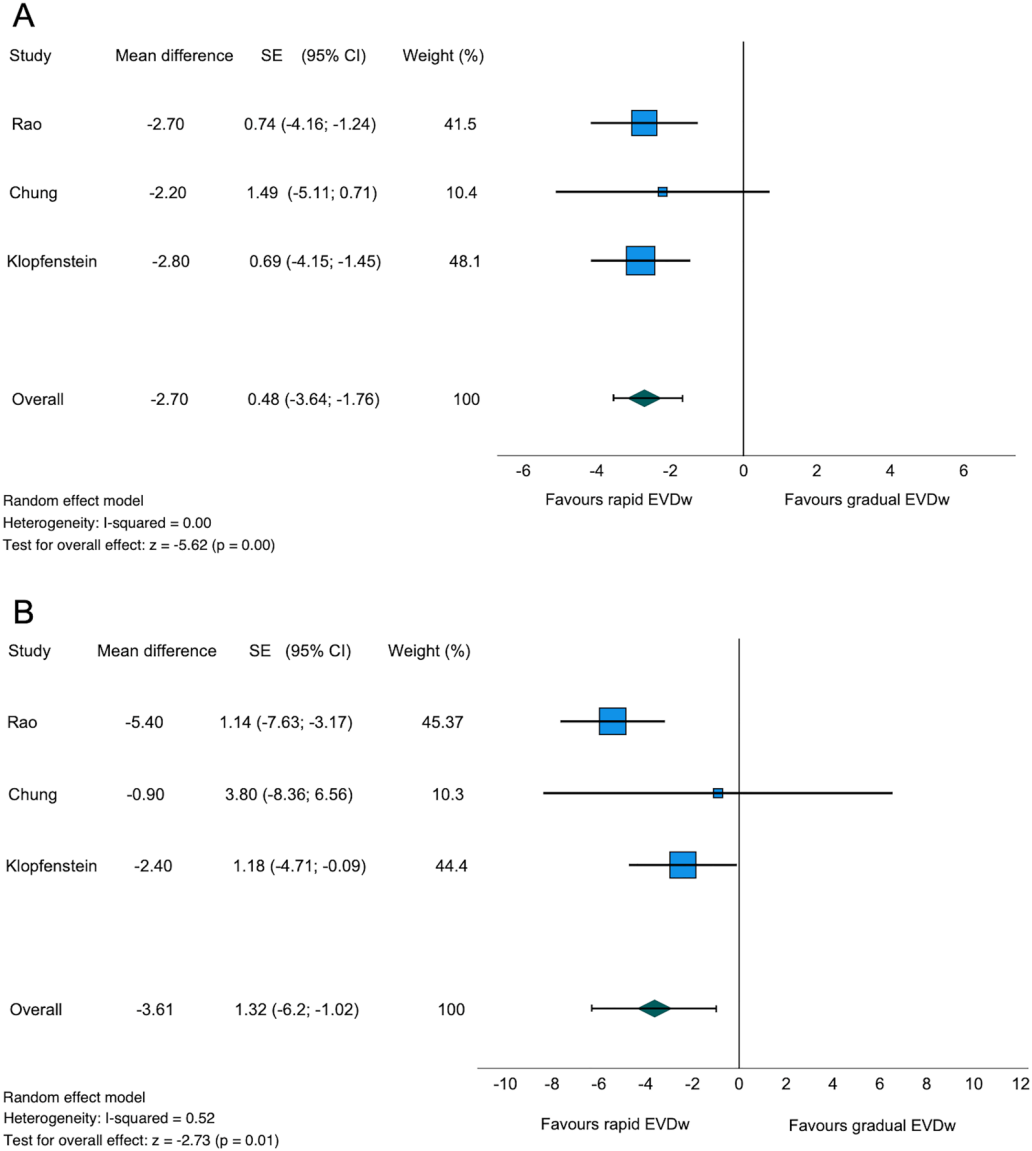
Forest plot of length of stay in the intensive care unit (**a**). Forest plot of total length of stay in the hospital (**b**). *CI* confidence interval, *EVDw* external ventricular drain weaning, *SE* standard error (standard deviation)

A total of three studies (75%) with 372 patients reported the ICU and total LOS [11, 13, 14]. The mean ICU LOS was 15.6 and 18.1 days in the rapid and gradual weaning groups, respectively (Table 2). The retrospective study by Jabbarli et al. [12] with 965 patients did not report the ICU LOS. Of the 372 patients, 220 patients (59.1%) were included from prospective studies, and 152 patients (40.9%) were from a retrospective study. The ICU LOS was significantly shorter in the rapid compared with the gradual weaning group (mean difference − 2.7 days, standard deviation 0.48, 95% CI − 3.64 to − 1.76, *I*^2^ = 0%, *z* =  − 5.62, *p* < 0.01, Table 2, Fig. 4a).

Similarly, a significantly shorter total LOS in patients with rapid EVD weaning compared with the gradual weaning group with a mean of 21.2 and 24.1 days was seen (mean difference − 3.61 days, standard deviation 1.32, 95% CI − 6.2 to − 1.02, *I*^2^ = 52%, *z* =  − 2.73, *p* = 0.01, Table 2, Fig. 4b) [11, 13, 14].

### Quality Assessment of Studies

**Fig. 5:**
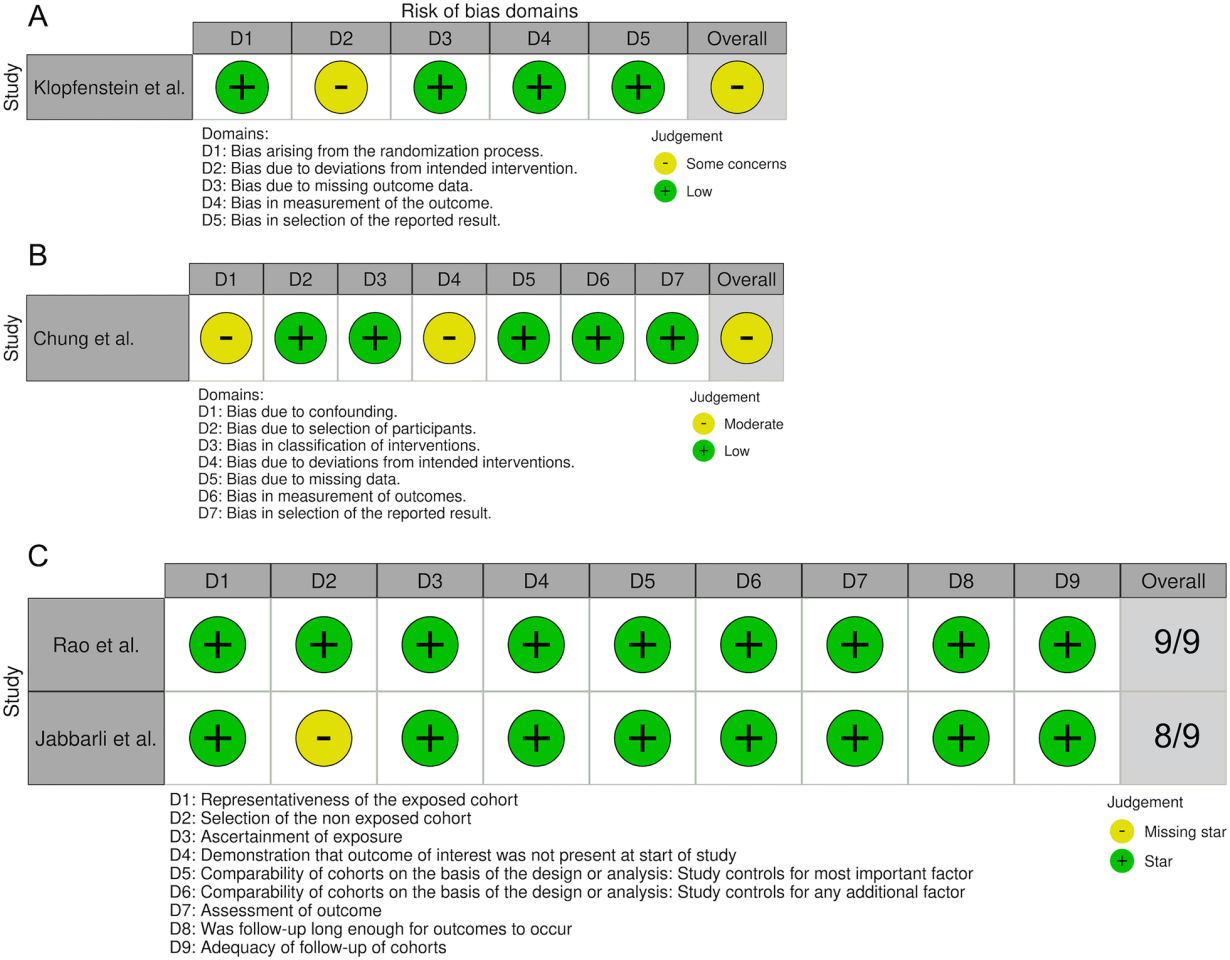
Traffic-light-plot depicting the quality assessment of the prospective randomized trial using the risk of bias tool for randomized trials (**a**), the prospective cohort study using the Risk of Bias in Nonrandomized Studies—of Interventions (ROBINS-1) (**b**), and the retrospective cohort studies using the Newcastle–Ottawa Scale (NOS) (**c**)

Of the four studies included, one was a RCT [14]. Based on the risk of bias tool, the RCT showed some concerns regarding the risk of bias due to the absence of blinding. Only the treating physician was blinded, which is why other involved caregivers and the patient himself might have known about the assignment to the respective treatment group [14]. Three studies were prospective or retrospective cohort studies [11–13]. Based on the Robins-1 tool, the risk of bias for the prospective cohort study was moderate due to potential confounding because in one of the six centers, the decision on which weaning method to perform was made by the treating physician, and due to the high crossover rate from gradual to rapid EVD weaning of 21% [13]. The two retrospective cohort studies were considered to have low risk of bias according to the NOS tool [11, 12]. In the study by Jabbarli et al. [12], the two treatment groups belonged to different centers, which is why 8 out of 9 points could be attributed with the NOS tool. The quality assessment of the studies is shown in Fig. 5.

## Discussion

The aim of this meta-analysis was primarily to investigate whether a difference in VPS insertion rate exists between gradual and rapid EVD weaning strategies. Secondarily, the EVDAI rate and the LOS in the ICU and the hospital were analyzed. The detected studies comparing gradual and rapid EVD weaning all included patients with SAH. Our meta-analysis showed no difference in VPS insertion and EVDAI rate, but patients receiving gradual EVD weaning stayed on average 2.7 days longer in the ICU and 3.6 days longer in the hospital than patients receiving rapid EVD weaning.

### Ventriculoperitoneal Shunt Insertion

Although EVD insertion is one of the most common emergency neurosurgical interventions, the overall evidence regarding optimal EVD management and weaning technique is low [19]. A common assumption is that gradual EVD weaning, with a slight decrease in CSF drainage stepwise, reduces the risk of VPS insertion. The hypothesis would be that the brain can slowly accommodate a new situation in which it needs to take over the resorption of CSF instead of the EVD. If this is done slowly and gradually, the success rate might be higher. However, this hypothesis could not be affirmed based on the presented results. One small RCT published in 2004 compared gradual and rapid EVD weaning in 81 patients and found no difference in VPS insertion rates, whereas the VPS rates within this study were very high (62.5% and 63.4%, respectively) [14]. Accordingly, the Neurocritical Care Society recommended in 2016 that an EVD should be weaned as fast as possible to reduce the risk of infection [20]. Nevertheless, a survey showed that gradual weaning of EVD is still much more frequently used (78%) than rapid weaning (22%) [21]. This implies insufficient conviction based on the available evidence recommending rapid EVD weaning.

Our analysis of 1337 patients showed an overall VPS insertion rate of 30%, which was lower than the RCT mentioned above but still higher than the VPS insertion rate otherwise mentioned in the literature [5]. Further, based on our meta-analysis, a comparable VPS insertion rate for gradual and rapid EVD weaning was found. However, high heterogeneity (*I*^2^ = 88%) between the included studies was seen. The included study by Rao et al. [11] retrospectively compared intermittent drainage and rapid EVD weaning with continuous drainage and gradual EVD weaning in a total of 152 patients suffering from SAH, finding a significantly lower VPS insertion rate in the rapid (13%) compared with the gradual EVD weaning group (35%). Two retrospective noncomparative cohort studies reported similar VPS insertion rates of 30–40.4% following gradual EVD weaning in patients suffering from SAH [15, 17]. The retrospective bicentric observational study by Jabbarli et al. [12], which analyzed 965 patients, showed a significantly lower VPS insertion rate in the gradual EVD weaning (27.5%) compared with the rapid EVD weaning group (34.7%). On the contrary, a prospective multicenter observational study by Chung et al. [13], analyzing 139 patients, as well as the RCT mentioned above, found no significant difference in the VPS rate between gradual and rapid EVD weaning [14]. A meta-analysis published by Palasz et al. [19] showed similar VPS rates between rapid and gradual EVD weaning strategies in patients treated with EVD after aSAH. Our meta-analysis includes an additional prospective study by Chung et al. [13] published recently. Overall, based on the available data, the rate of VPS after gradual or rapid EVD weaning is comparable. It is important to emphasize that the decision of whether to insert a VPS is usually based on the neurological condition and a computed tomography scan of the brain, and no standardized measurements exist to accurately predict when a VPS is required. However, multiple factors apart from the weaning strategy might affect the success of the weaning attempt, such as age, daily EVD output, the severity of the hemorrhage, and the amount of previous wean failures [22, 23]. These factors are not or inconsistently reported in the literature of this meta-analysis and may have influenced the decision of which weaning method was chosen in the retrospective studies, which may contain a potential bias. To further assess the impact of these factors, we encourage their inclusion in future prospective studies. Currently, an RCT, the DRAIN trial (NCT03948256), is in the recruitment phase with the goal of randomly assigning 244 patients with aSAH into rapid versus gradual weaning of the EVD, which will hopefully provide us with better evidence in the future to guide EVD management.

### External Ventricular Drain-Associated Infection

One of the most common complications associated with EVD is EVDAI. These occur in 9–20% of all patients with an EVD, with an incidence of 11 per 1000 catheter days [3, 15, 24, 25]. Secondary retrograde infection through the distal end of the EVD is the most common cause of infection [26]. Known risk factors for the development of EVDAI include long EVD duration, frequency of CSF sampling, continuous CSF drainage, and CSF leakage at the EVD’s entry site [27–29]. In our descriptive analysis, 11.4% of 1337 patients showed an EVDAI. In the meta-analysis, EVDAI rates did not differ significantly between the gradual and rapid EVD weaning groups (11.2% and 11.5%, respectively, *p* = 0.45). In the retrospective study by Jabbarli et al. [12], there was even a tendency toward a higher EVDAI rate of 15.33% in the rapid weaning group, compared with an EVDAI rate of 12.94% in the gradual weaning group. This finding is unexpected because gradual EVD weaning potentially leads to longer EVD duration and, thus, one would expect higher EVDAI rates. We suspect that patients with EVDAI may have been weaned more rapidly due to the infection. Because most (88.9%) of the included studies analyzed data retrospectively, this potential influence of EVDAI on the chosen weaning strategy was not assessed and therefore might skew the results. In the included RCT, EVDAI was not reported in either group [14]. The heterogeneity in our study was relatively high between the studies included in the meta-analysis (*I*^2^ = 57%). Therefore, our results need to be interpreted with caution.

### Length of Stay

Our descriptive analysis of the included studies showed a 2.5-day and 2.9-day reduction in ICU and hospital LOS in patients with rapid EVD weaning, respectively. The total hospital and ICU LOS in our meta-analysis differed significantly between the two weaning strategies. All studies included in the meta-analysis showed a clear trend in favor of rapid EVD weaning (Fig. 4a, b). The heterogeneity in our meta-analysis regarding ICU LOS as well as hospital LOS was low to moderate (*I*^2^ = 0% and *I*^2^ = 52%, respectively). This observation is expected because patients with EVD usually cannot be kept in a regular ward and often require intensive care. Because of the rising costs in the health care system, this observation is also of socioeconomic importance, as a stay in intensive care costs on average 1383 ± 398 euros per day and varies between European countries (1168–2025 euros per day) [30]. Overall, ICU costs account for one third of total health care costs [31]. In addition to the higher direct costs, longer stays in the ICU increase the risk of complications, such as the development of delirium or infections, leading to further increases in costs [32]. In our meta-analysis, 99.3% of the patients in the included studies suffered from aSAH. In contrast to patients after traumatic brain injury, patients with aSAH are at risk of vasospasm, which lasts up to 14 days [33]. Therefore, our study’s average hospital and ICU LOS is probably somewhat higher than in patients requiring EVD for other reasons.

In summary, ICU LOS and total hospital LOS are the only endpoints in which the included studies show consistent results. Rapid EVD weaning leads to significantly shorter LOS than gradual EVD weaning.

## Limitations

This article comprises some limitations. First, only two studies included in this analysis are of prospective nature, limiting the quality of the results [13, 14]. In both prospective studies, the study population is small. The remaining included studies are retrospective and therefore carry the limitations associated with such study design. Second, because of the lack of data, we could not analyze potentially influential factors, such as “time to first weaning attempt,” “total EVD duration,” “number of weaning attempts,” “EVD level upon weaning,” “CSF volumes drained until weaning”; CSF values, e.g., amount of red blood cells, proteins; and radiological parameters, such as the severity of intraventricular hemorrhage. These factors may have influenced our endpoints and therefore present a potential bias. Third, because of missing data, we could not analyze functional outcomes as measured by the modified Rankin Scale. Fourth, our study did not consider different EVD drainage methods, such as continuous or intermittent drainage. However, a previously published meta-analysis showed that this factor did not influence the VPS insertion rates [19].

## Conclusions

Based on the current data, gradual and rapid EVD weaning lead to comparable VPS insertion and EVDAI rates. ICU and hospital LOS are significantly shorter in patients with rapid EVD weaning. Therefore, rapid EVD weaning seems superior to gradual EVD weaning. Large RCTs need to confirm these results before clear recommendations can be made.

## Supplementary Information

Below is the link to the electronic supplementary material.

Supplementary file1 (DOCX 13 kb)
