# Supplementary material for: Rapid Versus Gradual Weaning of External Ventricular Drain: A Systematic Literature Review and Meta-analysis
Source: Neurocrit Care. 2023 Jun 12;39(1):250–9. doi: 10.1007/s12028-023-01766-6 (PMC10499951; doi:10.1007/s12028-023-01766-6)
Supplement: Supplementary file 1 — Supplementary file1 (DOCX 13 kb) [file 12028_2023_1766_MOESM1_ESM.docx]

**Supplementary Table 1.** Search terms for Pubmed, Embase, Web of Science.

| Database | Search terms |
| --- | --- |
| Pubmed | ("weaning"[Title/Abstract] OR "removal"[Title/Abstract] OR "remove"[Title/Abstract]) AND ((((EVD[Title/Abstract]) OR (external ventricular drain[Title/Abstract])) OR (ventriculostomy[MeSH Terms])) OR (ventriculostomy[Title/Abstract])) |
| Embase | (weaning:ab,ti OR 'device removal':ab,ti OR remove:ab,ti) AND (evd:ab,ti OR 'external ventricular drainage':ab,ti OR ventriculostomy:ab,ti OR 'ventriculostomy catheter':ab,ti) |
| Web of Science | (((((((TI=(EVD)) OR AB=(EVD)) OR TI=(external ventricular drain)) OR AB=(external ventricular drain)) OR AB=(ventriculostomy)) OR TI=(ventriculostomy))) AND ((((((AB=(weaning)) OR TI=(weaning)) OR TI=(removal)) OR AB=(removal)) OR AB=(remove)) OR TI=(remove)) |
